# Transcription of Nanofibrous Cerium Phosphate Using a pH-Sensitive Lipodipeptide Hydrogel Template

**DOI:** 10.3390/gels3020023

**Published:** 2017-06-10

**Authors:** Mario Llusar, Beatriu Escuder, Juan de Dios López-Castro, Susana Trasobares, Guillermo Monrós

**Affiliations:** 1 Departamento de Química Inorgánica y Orgánica, ESTCE, Universitat Jaume I, Av. de Vicent Sos Baynat s/n, 12071 Castellón de la Plana, Spain; escuder@qio.uji.es (B.E.); monros@uji.es (G.M.); 2 Departamento de Ciencia de los Materiales e Ingeniería Metalúrgica y Química Inorgánica, Universidad de Cádiz, c/República Saharahui s/n, Aptdo. 40, Puerto Real, 11510 Cádiz, Spain; juan.lopezcastro@uca.es (J.d.D.L.-C.); susana.trasobares@uca.es (S.T.)

**Keywords:** transcription, templating, hydrogel, cerium phosphate nanofibers, hexagonal rhabdophane, monazite, FE-SEM, TEM/HRTEM, STEM-HAADF, UV-photo-luminescence

## Abstract

A novel and simple transcription strategy has been designed for the template-synthesis of CePO_4_·xH_2_O nanofibers having an improved nanofibrous morphology using a pH-sensitive nanofibrous hydrogel (glycine-alanine lipodipeptide) as structure-directing scaffold. The phosphorylated hydrogel was employed as a template to direct the mineralization of high aspect ratio nanofibrous cerium phosphate, which in-situ formed by diffusion of aqueous CeCl_3_ and subsequent drying (60 °C) and annealing treatments (250, 600 and 900 °C). Dried xerogels and annealed CePO_4_ powders were characterized by conventional thermal and thermogravimetric analysis (DTA/TG), and Wide-Angle X-ray powder diffraction (WAXD) and X-ray powder diffraction (XRD) techniques. A molecular packing model for the formation of the fibrous xerogel template was proposed, in accordance with results from Fourier-Transformed Infrarred (FTIR) and WAXD measurements. The morphology, crystalline structure and composition of CePO_4_ nanofibers were characterized by electron microscopy techniques (Field-Emission Scanning Electron Microscopy (FE-SEM), Transmission Electron Microscopy/High-Resolution Transmission Electron Microscopy (TEM/HRTEM), and Scanning Transmission Electron Microscopy working in High Angle Annular Dark-Field (STEM-HAADF)) with associated X-ray energy-dispersive detector (EDS) and Scanning Transmission Electron Microscopy-Electron Energy Loss (STEM-EELS) spectroscopies. Noteworthy, this templating approach successfully led to the formation of CePO_4_·H_2_O nanofibrous bundles of rather co-aligned and elongated nanofibers (10–20 nm thick and up to ca. 1 μm long). The formed nanofibers consisted of hexagonal (P6_2_22) CePO_4_ nanocrystals (at 60 and 250 °C), with a better-grown and more homogeneous fibrous morphology with respect to a reference CePO_4_ prepared under similar (non-templated) conditions, and transformed into nanofibrous monoclinic monazite (P21/n) around 600 °C. The nanofibrous morphology was highly preserved after annealing at 900 °C under N_2_, although collapsed under air conditions. The nanofibrous CePO_4_ (as-prepared hexagonal and 900 °C-annealed monoclinic) exhibited an enhanced UV photo-luminescent emission with respect to non-fibrous homologues.

## 1. Introduction

Rare-earth phosphates (from now on, *RPO_4_*) are being prolifically investigated during the last decades given the wide variety of interesting properties they may exhibit, such as optical, electronical (or optoelectronical), ion-exchange, catalytic, heat-resistance, and biocompatibility, among others. These advanced properties have enabled the application of *RPO_4_*, especially CePO_4_ and related solid solutions (with Tb, Gd, or other lantanides) and composite systems (Au/CePO_4_,...) in a plethora of technological applications, such as optoelectronic devices (luminescent materials, phosphors, displays, green light-emitting diodes or switches, solid-state lasers, redox sensors, non-linear optical devices,...) [1,2,3,4,5,6,7,8,9,10,11,12,13], fluorescent probes for chemical sensing [14,15,16] and detection or removal of heavy metals (e.g., Pb^2+^, Co^2+^,...) [17,18], bio-sensing, bio-imaging and cell-labeling applications [19,20,21,22,23], ceramic materials (with high thermal and mechanical properties) [24], dielectrics [25], catalysts [26,27,28,29,30], ion-exchange or ion-conducting materials (proton-conducting membranes) for solid-oxide fuel cells (SOFCs) [25,31] and solar-cells, ceramic pigments [32,33,34], UV filters for sunscreens [35], and so on.

It is well-known that one-dimensional (1D) nanostructured materials may exhibit improved or even novel properties with respect to their bulk counterparts, due to quantum-sized nano-confinement effects, becoming especially suited to design better performant electronic, optoelectronic, electrochemical or electromechanical devices [36,37]. In the case of lanthanide phosphate materials, e.g., CePO_4_ and related solid solutions, composite systems and core–shell heterostructures, the preparation of highly-anisotropic 1D nanostructures with different morphologies (such as nanowires, nanorods, nanobelts, nanotubes, nanobars, nanocables, spindle-like, etc.) has led to highly-efficient and even better-performant photoluminescent, optoelectronic or electrochemical devices, sensors and biomaterials [1,2,3,4,5,6,9,11,15,16,20,21,22,38,39,40,41,42,43,44,45,46,47,48,49,50,51].

Recent investigations have also evidenced that the properties of CePO_4_ nanoarchitectures depend strongly on its dimensionality, morphology, size, shape and size uniformity and crystallinity (structure- and morphology-properties relationships), and these aspects are very sensitive to the employed synthesis methodology [1,3,4,5,6,7,8,12,13,41,48,49,50,51,52,53,54,55,56]. In this respect, although, 1D *RPO*_4_ nanomaterials like CePO_4_ may be prepared by relatively facile or simple solution methods [4,52,54], the controlled synthesis of these nanomaterials often requires the employment of non-conventional routes such as sol-gel [24,34], hydrothermal [3,5,7,38,39,41,43,48,49,57,58,59], microemulsion [45,60], sonochemical [18,51,61,62], or microwaves-assisted methods (combined or not with hydrothermal or solvothermal treatments) [9,22,42,56]. In these methods, the addition of chelating or complexing additives and surfactants is often necessary [1,5,7,30,45,55,63], and a rigorous and judicious control of synthesis conditions is generally needed to obtain the desired morphology, including preparation variables such as the chosen Ce and phosphate sources, the P/Ce ratio, reaction temperature and time, pH and ageing conditions, the order and speed of precursors addition (rapid vs. dropwise or continuous-flow) [4,6,7,10,12,18,41,48,52,53,54,56], and so on. For instance, many studies have revealed the need of using high P:Ce ratios (much higher than 1:1) or very acidic conditions (pH values < 1.5) to obtain more uniform and highly-elongated (high aspect ratio) morphologies with a better crystallinity, and these characteristics become essential to improve the photoluminescent properties of CePO_4_ [4,39,41,48,52,54,57].

The employment of template-based synthesis strategies could be also a relatively simple and alternative way to obtain highly anisotropic and better performant CePO_4_ nanofibrous materials. However, to the best of our knowledge, only scarce precedents are reported in the literature on the template-assisted synthesis of *RPO*_4_ 1D nanostructures [20]. For this purpose, highly anisotropic (nanofibrous) organogel or hydrogel templates could be especially suited, since they have been already employed satisfactorily as nanofibrous templates to direct the mineralization of a wide variety of advanced 1D inorganic or hybrid inorganic-organic nanomaterials [64,65,66,67,68,69,70,71,72,73]. Among the different families of low-molecular-weight gelators, amino acid-based derivatives (e.g., based on valine, lysine, glycine or alanine peptides, dipeptides or tetrapeptides) have proven to be ideal candidates to obtain nanofibrous pH-sensitive (or pH-responsive) universal gelators [74,75,76,77,78,79,80,81,82]. In addition, peptide or lipopeptide amphiphiles may be also easily functionalized to become reactive or functional gels [83], and they have been also successfully employed as templates in the transcription of nanofibrous inorganic materials and hybrid biomaterials (see e.g., references [73,74,84,85,86,87,88]).

**Scheme 1:**
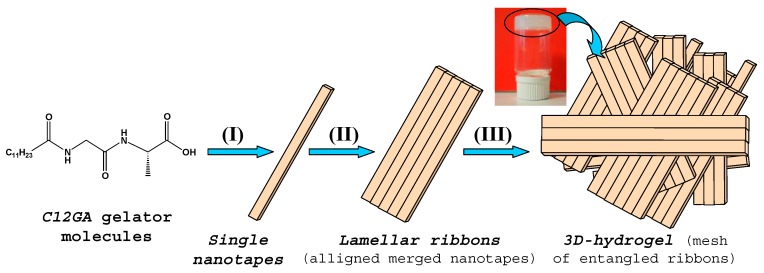
Proposed scheme for the hierarchical self-assembly of lipodipeptide C12GA gelator (C_17_H_32_N_2_O_4_, *N*-Dodecanoyl-glycyl-l-alanine) in aqueous NaH_2_PO_4_ media: gelator molecules self-assemble into lamellar-like elongated nanotapes (**I**). The single nanotapes merge into lamellar ribbons (**II**), which entangle and collapse forming the 3D-hidrogel (**III**).

**Scheme 2:**
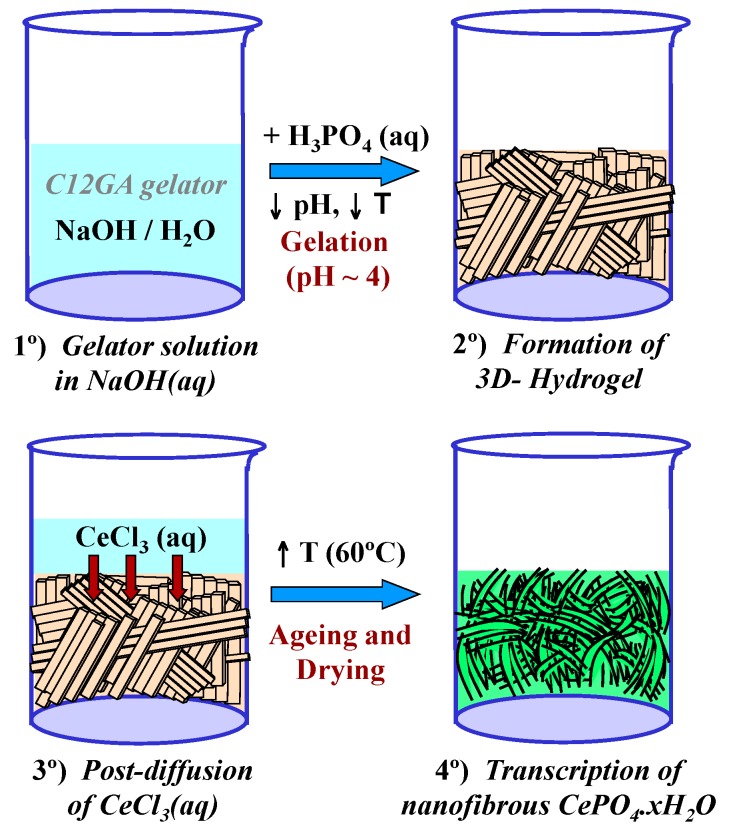
Transcription strategy (post-diffusion) for the mineralization of nanofibrous CePO_4_·H_2_O through the use of a preformed hydrogel template of phosphorylated C12GA.

With the above precedents, in the present investigation, we chose a lipodipeptide derivative [88] (*N*-acyl glycine-alanine, C12GA, see Scheme 1-left) as pH-sensitive hydrogelator. This gelator is easily solved in basic media (e.g., in aqueous NaOH), and hydrogelation may be easily induced by softly reducing the pH with some acid (up to ca. 4.5) giving rise to a self-assembled fibrous 3D-hydrogel. Given the strong affinity of carboxylate or peptide moieties for phosphate species [84,86,89,90,91], it thus occurred to us that C12GA hydrogelation could also be induced by acidification with H_3_PO_4_, thus simultaneously incorporating (grafting) the precursor phosphate moieties throughout the self-assembled nanofibrous network (SAFIN). Then, the obtained phosphorylated C12GA hydrogel could be used as nanofibrous template to direct the mineralization of high aspect ratio nanofibrous CePO_4_ by post-diffusion (impregnation) with an aqueous Ce^3+^ solution (see the proposed transcription strategy later in Scheme 2, Section 2.2.1). As important advantage, this transcription strategy may be performed under relatively mild conditions (pH around 4–4.5) avoiding the strict requirements of using a great excess amount of phosphates (with detrimental environmental effects due to the presence of phosphates in washing waters) and also of very low pH values (<1.5), which are often necessary to obtain high aspect ratio elongated CePO_4_ nanofibers.

## 2. Results and Discussion

Herein, we describe the results obtained in the template-based synthesis of nanofibrous cerium phosphate employing a pH-sensitive nanofibrous hydrogel as structure-directing scaffold. The morphology, composition and crystal chemical characterization of the precursor hydrogel template and the resulting mineralized nanofibrous CePO_4_·xH_2_O have been performed through different solid-state characterization techniques, and compared with a non-templated CePO_4_ prepared under similar conditions. Moreover, the thermal stability, crystalline phase evolution and photoluminescence properties of the templated nanofibrous CePO_4_ (as-prepared and after annealing at 250, 600 and 900 °C) are also analyzed.

### 2.1. Characterization of Self-Assembled Nanofibrous Hydrogel Template

#### 2.1.1. Aggregation Behavior and Morphology (SEM/EDX + TEM) of Nanofibrous C12GA Hydrogel

Gelation tests performed with C12GA (and other related glycine-alanine lipodipeptide derivatives) showed that C12GA did not form gels and remained soluble in polar (alcohols) and apolar solvents at concentrations of 5 or even 10 mg/mL, although organogelation was attained at slightly higher concentrations. However, translucent or transparent organogels could be formed in different organic solvents at a relatively low concentration of 5 mg/mL (15 mM) after placing in gaseous chambers of HCl (owing to salt formation or protonation of carboxylic group) [92].

**Figure 1:**
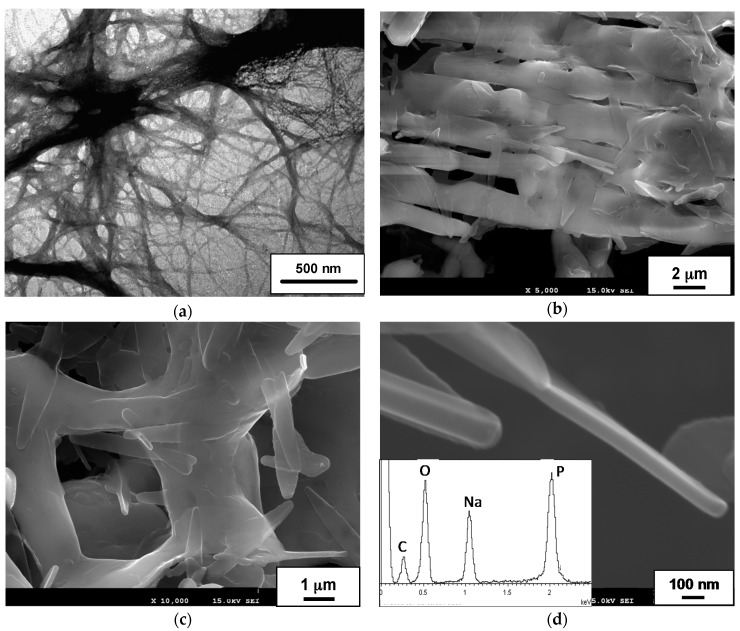
(**a**) TEM image of HCl-C12GA hydrogel (Pt-shadowing) formed in aqueous acidic medium (HCl vapors); (**b**–**d**) SEM images of H_3_PO_4_-C12GA hydrogel in aqueous NaH_2_PO_4_ medium (lyophilized xerogel). The inset in (**d**) shows a representative EDX spectrum of the NaH_2_PO_4_-containing C12GA xerogel (average P/Na molar ratio = 1.19 ± 0.07).

Regarding to its hydrogelation behavior, C12GA lipodipeptide readily formed hydrogels in acidic aqueous media (pKa value of C12GA: ca. 4.5), being the hydrogel morphology very sensitive to gelator concentration and gelation protocol. For instance, using the general gelation procedure (free cooling to room temperature), an opaque hydrogel formed at very low concentrations (5 mg/3.5 mL, or 4.35 mM), predominantly formed by micron-sized, large and rigid tapes (not shown) [92]. In contrast, at a slightly higher concentration of 15 mM (5 mg dissolved in 1 mL of 0.1 M NaOH) and inducing gelation by soft acidification through exposure to HCl vapors, C12GA compound was able to form a 3D hydrogel network (HCl-C12GA hydrogel) consisting of a mesh of entangled fibrous bundles (100–250 nm width) formed by smaller nanofibrils (of even less than 20 nm), as it is shown in the TEM image of Figure 1a.

In order to obtain a phosphorylated C12GA hydrogel (H_3_PO_4_-C12GA hydrogel) to be used as nanofibrous scaffold for the transcription of CePO_4_ nanofibers, hydrogelation of C12GA compound was induced by acidification with aqueous H_3_PO_4_, thus incorporating the phosphate moieties throughout the self-assembled hydrogel network. As a consequence of the higher salinity conditions of the initial basic (NaOH) solution of C12GA gelator with respect to HCl-hydrogel (NaOH:C12GA molar ratio of 33:1 against 6.6:1), along with the presence of H_2_PO_4_^−^ species able to interact with carboxylate and amide moieties of C12GA, the self-assembled phosphorylated hydrogel exhibited in this case a different morphology. As it may be appreciated in the SEM images of Figure 1b–d, the dried (lyophilized) H_3_PO_4_-C12GA xerogel consisted of a mesh of long nanoribbons or nanotapes (60–100 nm width, 100–140 nm thick, and hundreds of microns length), which merged laterally forming broader tapes (up to 3–5 microns width) and a relatively branched or entangled 3D-network. According to the model shown in Scheme 1, it seems that C12GA gelator molecules self-assemble into lamellar-like elongated single nanotapes (I), which merge into broader lamellar ribbons (II), and then these lamellar ribbons branch forming a rather entangled 3D-hidrogel (III).

#### 2.1.2. Crystal-Chemical Characterization (by WAXD and FTIR) of Hydrogel Template

**Figure 2:**
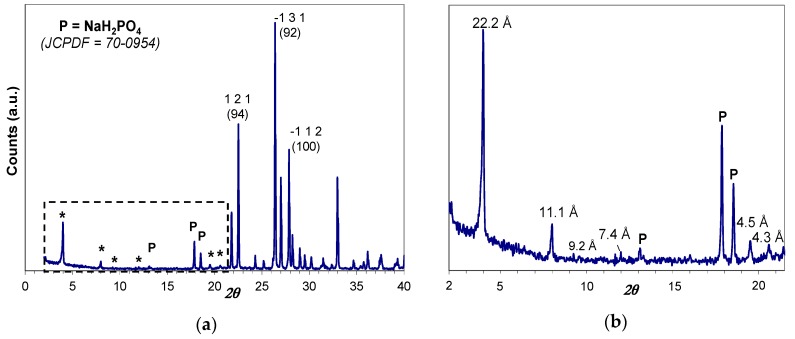
XRD patterns of the NaH_2_PO_4_-based C12GA xerogel: (**a**) overall pattern; and (**b**) magnification of the low-angle region marked with the dashed rectangle. The peaks labelled with an asterisk correspond to the periodical arrangement of C12GA gelator molecules (the distances are shown in the magnification), and all the remaining peaks correspond to NaH_2_PO_4_ phase (monoclinic P21/c space group, JCPDF number 70-0954) marked as **P** only in the low angle region; the Miller indexes and theoretical relative intensities of the three most intense peaks of NaH_2_PO_4_ are indicated.

The as-prepared hybrid H_3_PO_4_-C12GA xerogel (dried, lyophilized hydrogel) was characterized by Wide-Angle X-ray powder diffraction technique (WAXD), which also covers the low-angle region between 2° and 20° 2θ. As it may be appreciated in Figure 2, the obtained XRD patterns revealed the crystalline nature of as-prepared xerogel, exhibiting intense peaks corresponding to the crystallization of anhydrous NaH_2_PO_4_ (monoclinic P21/c space group, JCPDF number 70-0954). The presence of crystalline NaH_2_PO_4_ forming part of the self-assembled 3D hydrogel arises from the neutralization of H_3_PO_4_ by NaOH occurring in the hydrogelation procedure (aqueous H_3_PO_4_ was added to an aqueous NaOH solution of C12GA to induce hydrogelation, with a H_3_PO_4_:NaOH molar ratio of 1.1:1; see details in Section 4.1); moreover, H_2_PO_4_^−^ moieties may also arise from the protonation of amide and carboxylate moieties of C12GA molecules by H_3_PO_4_.

Noteworthy, several and relatively intense diffraction peaks may be also observed in the low-angle region below 20° 2θ (marked with and asterisk), which are associated with the long-range ordering or periodic packing (3D self-assembly) of C12GA gelator molecules in the nanofibrous hydrogel. The main and sharper peaks at lower angles around 4° and 8° 2θ correspond to d spacings of 22.2 and 11.1 Å, respectively, which fit very nicely with the whole and half calculated distances for an extended C12GA gelator molecule, and the less intense peak around 12° 2θ (7.4 Å spacing) might be also assigned to the third order diffraction of this 22.2 Å distance. Additionally, other relevant peaks of lower intensity are observed at wider angles corresponding to 4.5 and 4.3 Å spacings (and also to 9.2 Å, with a much lower intensity), which could be ascribed to the two-dimensional packing of sheet-like structures. A proposed packing or aggregation model for C12GA molecules in the self-assembled (phosphorylated) hydrogel is later discussed (see Section 2.1.3), in accordance with the features observed in this low-angle region of the WAXD pattern.

**Figure 3:**
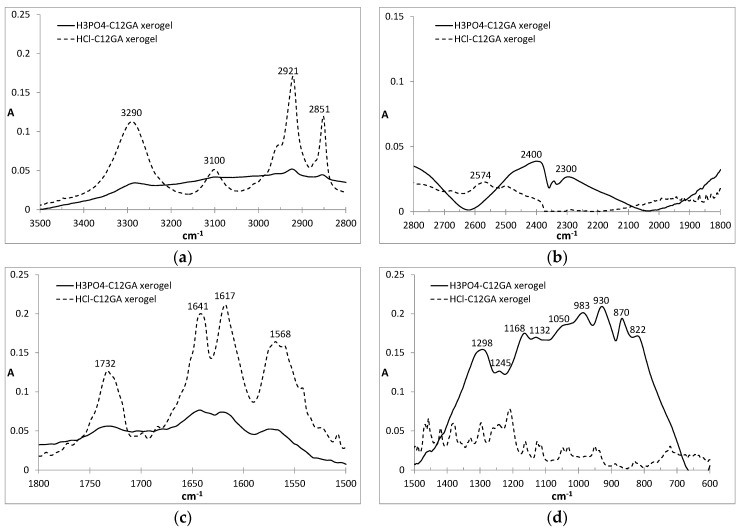
FTIR spectra of C12GA dried xerogels obtained in acidic media: HCl (dashed line), H_3_PO_4_ (solid line): (**a**) 3500–2800 cm^−1^; (**b**) 2800–1800 cm^−1^; (**c**) 1800–1500 cm^−1^; (**d**) 1500–600 cm^−1^.

FTIR is also a useful technique for the study of molecular aggregates and, in particular, to detect the participation of H-bonding in the aggregation process [93]. In our case, the FTIR spectra of HCl-C12GA and phosphorylated H_3_PO_4_-C12GA xerogels, both of them prepared by liophilization of the corresponding hydrogels formed by pH change (the former through exposure to HCl vapors and the later by the addition of aqueous H_3_PO_4_) are compared in Figure 3. Both xerogels revealed a relatively similar FTIR pattern, although with considerably broader and less-defined bands for the last one. The C=O stretching region showed several bands corresponding to both carboxyl and amide groups. Carboxyl groups may exist in three different environments depending on the H-bonding interactions in which they are involved: free monomer (1730 cm^−1^), intermolecular acyclic dimmers (1720 cm^−1^) and intermolecular cyclic dimmers (1700 cm^−1^) [94]. As can be seen in Figure 3, a band centered at ca. 1730 cm^−1^ appears in both xerogels suggesting the presence of non H-bonded carboxyl groups. On the other hand, the amide I stretching band is also sensitive to the presence of H-bonding. As can be seen in Figure 3, two different peaks at 1617 and 1641 cm^−1^ can be observed suggesting that one of the amide C=O has a strong H-bonding and the second one only moderate, respectively. The first band corresponds to the Gly amide that is located close to the hydrophobic tail which favors strong H-bonding, whereas L-Ala amide H-bonding strength is reduced as it is in a more polar environment at the proximity of the carboxyl group [95]. Amide II vibrations appear as a broad band centered at 1568 cm^−1^, in agreement with the participation in H-bonding. On the other hand, the N-H vibration region also revealed interesting results: in the case of HCl-xerogel the characteristic broad band of an associated carboxylic acid is absent and an intense band centered at 3290 cm^−1^ involves both O–H and N–H vibrations. In the case of H_3_PO_4_-xerogel, although similar peaks are present, a broad background suggests the possibility of additional H-bonding between O-H and presumably the entrapped NaH_2_PO_4_ salt. Additionally, intense bands appear at 2921 and 2851 cm^−1^ corresponding to anti-symmetric and symmetric CH_2_ stretching modes, respectively, indicating a tight packing of extended alkyl chains as a consequence of van der Waals interactions and the hydrophobic effect.

Moreover, the FTIR pattern of the H_3_PO_4_-xerogel exhibits the characteristic broad band in the low energy region (600–1500 cm^−1^) with multiple peaks from 800 to 1300 cm^−1^ associated to the vibrations of PO_2_ and P(OH)_2_ groups present in H_2_PO_4_^−^ species [96]. Indeed, the positions of the peaks are very similar to those of anhydrous NaH_2_PO_4_ [97], with some of the peaks slightly shifted presumably due to H-bonding interaction of phosphates with amine or amide groups of C12GA. The absence of the typical symmetric and anti-symmetric OH stretching vibration of water molecules at around 3500 cm^−1^ confirms also the formation of non-hydrated NaH_2_PO_4_.

#### 2.1.3. Proposed Model for the Packing of C12GA Gelator in Self-Assembled Hydrogel

**Figure 4:**
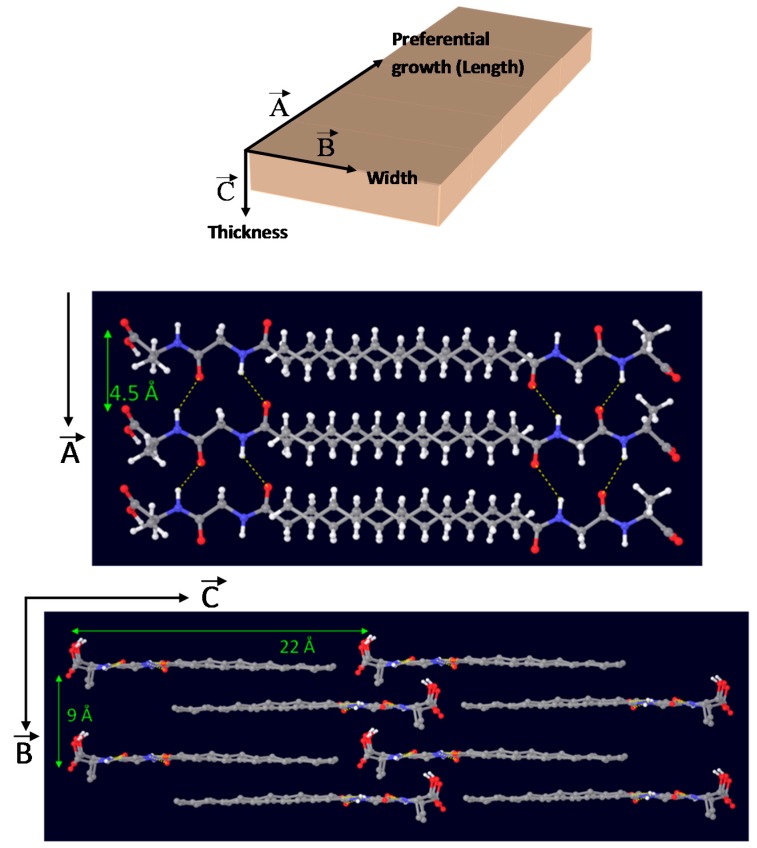
Energy-minimized structure models (MACROMODEL 7.0, AMBER* [98]) for the packing of compound C12GA in phosphorylated xerogel (non-polar hydrogens are omitted for clarity in the bottom image).

Taking together results from FTIR and WAXD characterization, an energy-minimized model for the 3D-packing of C12GA gelator molecules in the self-assembled nanofibrous xerogel can be proposed (Figure 4). In this model, arbitrary axes (A, B and C) have been assigned corresponding to the growing directions of the lamellar nanotapes. The preferential growth would be along A and B axes, responsible for the nanotapes length and width dimensions (associated with packing distances of 4.5 and 9 Å, respectively), while packing along C axis would be responsible of the nanotapes thickness (associated with the calculated 22 Å distance of extended C12GA compound). According to this model, the hydrophobic effect is the main driving force for aggregation creating a layered structure with a preferential 2D-growth that leads to the fibrous-like nanotapes observed by electron microscopy. H-bonding is also present, although it is not supposed to play a fundamental role in aqueous environment. In this model of aggregation carboxylic groups would be buried within hydrophobic pockets and isolated from other H-bonding groups as well as water molecules. An alternative energy-minimized packing model is also shown as Supplementary Figure S1.

### 2.2. Templated-Based Synthesis and Characterization of Hybrid C12GA-CePO_4_ Xerogel (60 °C)

#### 2.2.1. Transcription Strategy for the Preparation of C12GA-Templated Nanofibrous CePO_4_

Scheme 2 shows the transcription (post-diffusion) strategy employed for the mineralization of nanofibrous CePO_4_·xH_2_O through the use of a preformed hydrogel template of phosphorylated C12GA. As a related precedent, Hartgerink et al employed also a fibrous pH-sensitive peptide amphiphile as nanofibrous scaffold to direct the mineralization of hydroxyapatite [81]. As may be seen, hydrogelation was induced by acidification of C12GA basic solution with H_3_PO_4_ up to pH = ca. 4 (H_3_PO_4_:NaOH = 1.1:1) and rapid cooling in an ice bath (see details in Section 4.1). Then, this phosphorylated hydrogel was employed as a sheet-like nanofibrous scaffold to direct the mineralization of CePO_4_ (Section 4.2) which in-situ formed by post-diffusion of (or impregnation with) aqueous CeCl_3_, and soft drying at 60 °C (72 h/air). A similar post-diffusion transcription strategy was also satisfactorily employed to replicate silica nanotubes using another amino acid-based hydrogel as templating agent [73]. The as-obtained hybrid C12GA-CePO_4_ xerogel was subsequently washed (until complete NaCl removal) and annealed at different temperatures (250, 600 and 900 °C).

#### 2.2.2. Characterization of Templated Nanofibrous C12GA-CePO_4_ Xerogel (60 °C)

**Figure 5:**
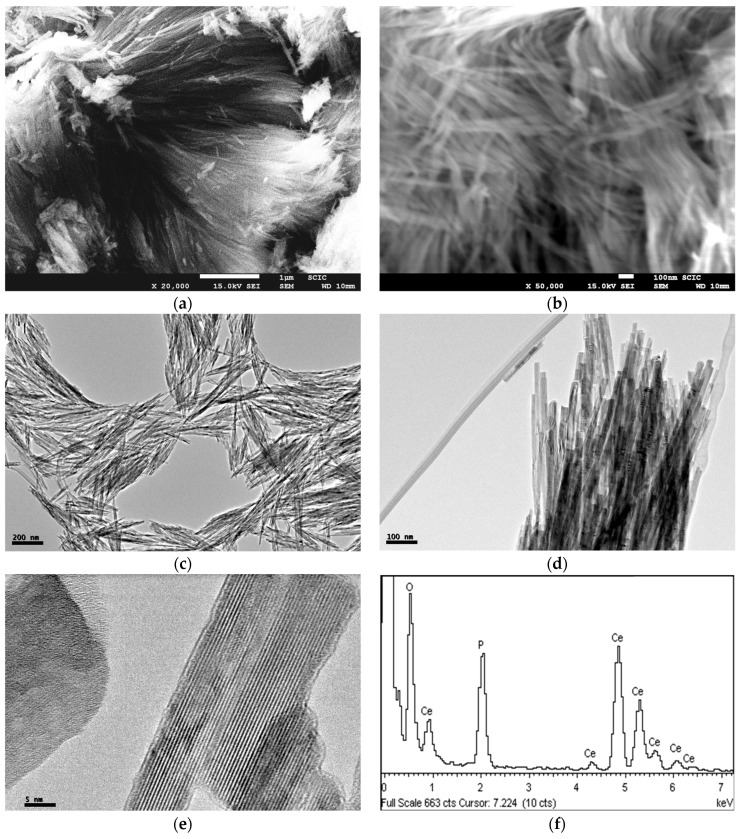
FE-SEM images (**a**,**b**), and TEM images (**c**–**e**) of the as-prepared C12GA-templated nanofibrous CePO_4_ (60 °C-dried and washed xerogel); (**f**) Representative energy-dispersive EDX spectrum of this CePO_4_ xerogel (60 °C).

Electron microscope images (FE-SEM and TEM) of the hybrid C12GA-CePO_4_ dried (60 °C) and washed xerogel prepared with the above transcription strategy are shown in Figure 5, while those of a reference CePO_4_ sample (60 °C-annealed) obtained under similar but non-templated synthesis conditions (see details in Section 4.3) may be seen in Figure S2. Noteworthy, the employed template-based synthesis method satisfactorily led to the formation of a mesh of fibrous bundles (Figure 5a,b), constituted by very thin, elongated and rather co-aligned (poorly entangled or inter-branched) high aspect-ratio nanofibrils around 20 nm thick and even above 1 micron length. A closer inspection of these nanofibrils with the help of higher magnification TEM images (Figure 5c,d) revealed a morphology based on rather straight and rod-like single or individual nanofibrils (ca. 10 nm thick and hundreds of nanometers length), partially and laterally merged forming slightly entangled sheet-like nanotapes (Figure 5d). These multi-fibril-based sheet-like nanotapes have some resemblance with the original mesh of nanoribbons or nanotapes observed in the H_3_PO_4_-C12GA xerogel template (see, e.g., FE-SEM image of Figure 1c), and could be a confirmation that CePO_4_ mineralization was effectively directed (templated) by the H_3_PO_4_-C12GA xerogel employed as nanofibrous scaffold. Indeed, it seems quite plausible that CePO_4_ mineralization may cause the disaggregation of the single nanotapes, which were previously merged forming broader and dense lamellar ribbons (several microns thick) in the original H_3_PO_4_-C12GA xerogel (Figure 1b,c and also Scheme 1), thus resulting into a mesh of straight and co-aligned single nanofibrils.

The higher magnification TEM image shown in Figure 5e confirms the single-crystalline character of these flat nanofibers (around 7–10 nm thick and laterally merged forming the multi-fibril nanotapes). Regarding to the chemical composition, EDS spectra recorded in different regions of these nanofibrous bundles (a representative EDS spectrum is shown in Figure 5f) gave an average P/Ce molar ratio of 1.14 ± 0.03, which is well correlated with the expected chemical composition according to the H_3_PO_4_:CeCl_3_ molar ratio employed in the synthesis (5.5:5 = 1.1).

The reference CePO_4_ sample prepared under similar (non-templated) synthesis conditions exhibited also nanofibrous morphology (Figure S2). However, this sample contained also amorphous regions with more rounded shapes, and the observed nanofibers were less homogeneous and shorter (lower aspect ratio) forming rather bulk aggregates. This morphology is the usually observed for CePO_4_ prepared when mixing H_3_PO_4_ and Ce^3+^ salts (chlorides or nitrates) without employing a high excess of phosphate species (P:Ce molar ratios above 2:1 or even higher) or highly acidic conditions (pH < 1.5). Therefore, it must be highlighted that the hydrogel-template strategy herein employed has satisfactorily led to the formation of more homogenous and elongated CePO_4_ nanofibrils under relatively mild conditions (P:Ce molar ratio very close to 1:1, and slightly acidic conditions with a pH of ca. 4).

**Figure 6:**
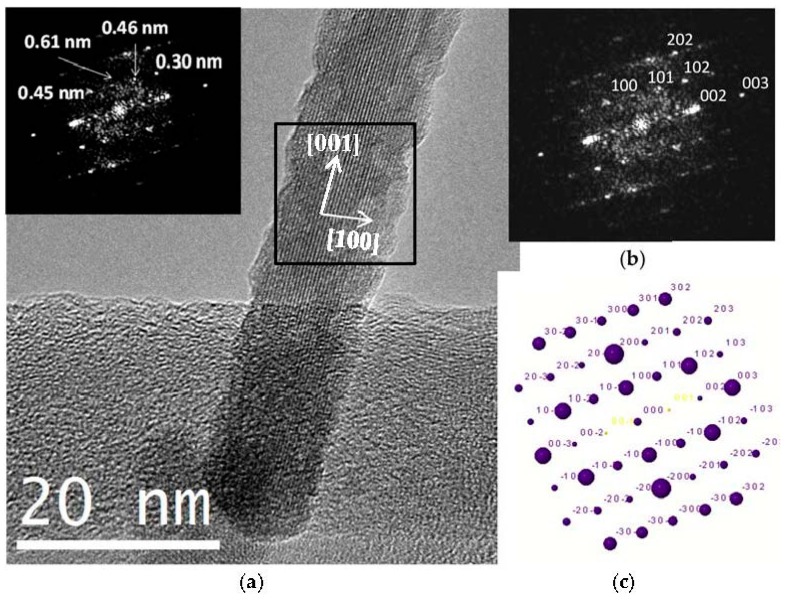
(**a**) HRTEM image of a representative nanofiber of as-prepared C12GA-templated nanofibrous CePO_4_ (60 °C-dried and washed xerogel), the inset showing the corresponding digital diffraction pattern (DDP) of the region marked with an square, with indicated distances; (**b**) same DDP with indexed (*hkl*) values (taken along zone axis [uvw]: [010]); (**c**) Corresponding simulated kinetic diffraction diagram.

A crystal chemical characterization by XRD technique of reference and C12GA-templated CePO_4_ samples (60 °C-annealed) confirmed the formation of hexagonal CePO_4_ (space group P6_2_22) in both cases (see later in Section 2.3.1). In the case of C12GA-templated sample, this was also confirmed by a more in-depth crystal chemical characterization through high-resolution transmission electron microscopy (HRTEM). Figure 6a shows the HRTEM image of a representative single-crystalline CePO_4_ nanofiber (around 10–15 nm thick). The corresponding digital diffraction pattern (DDP) obtained for the small area marked with a square (inset of Figure 6a) shows the typical diffraction spots, associated to inter-planar distances and angles (d1/d2/angle: 0.61/0.46/43.1, 0.61/0.30/61.5 and 0.61/0.45/43.8) that can only be assigned to hexagonal CePO_4_. The DDP of Figure 6b shows the assignment of some of these spots to the corresponding (hkl) lattice planes of the hexagonal system ([uvw] zone axis: [010]), and the corresponding simulated kinetic diffraction diagram is also shown in Figure 6c. The lattice fringes observed in Figure 6a would correspond to the (100) facets with a d-spacing of 0.61 nm, growing the nanofiber in the [001] direction.

**Figure 7:**
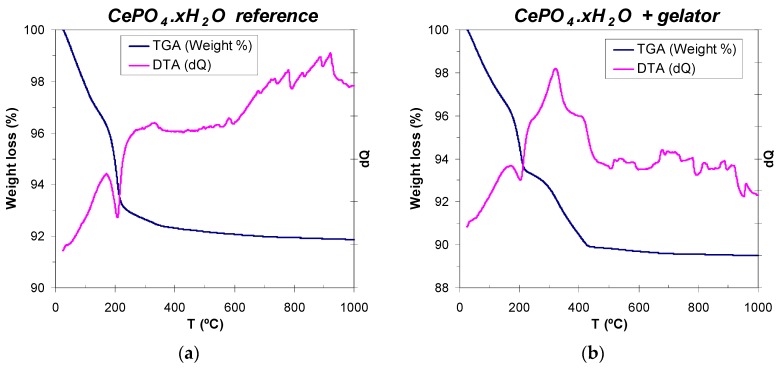
Differential thermal and thermogravimetric analysis (DTA-TGA) of as-prepared (60 °C-dried and washed) samples: (**a**) non-templated reference CePO_4_; and (**b**) templated CePO_4_ xerogel.

It is well-known that cerium phosphate crystallizes at low temperatures as an hydrated hexagonal phase (hexagonal rhabdophane, CePO_4_·xH_2_O, which usually contains one mol of hydration water, x = 1) [99,100], and that the evolution of water molecules to render anhydrous hexagonal CePO_4_ phase needs of annealing treatments at 200 °C or even up to 600 °C [24,25,99]. Therefore, thermal analysis (WAXD) of reference (non-templated) and C12GA-templated CePO_4_ was performed in order to determine the amount of hydration water present in 60 °C-dried samples, and also the temperature range for the removal (decomposition) of the self-assembled C12GA gelator scaffold in the case of templated CePO_4_. The reference CePO_4_ sample exhibits a three-step decomposition scheme (Figure 7a), with a first 2.5% weight loss between 25 and 110 °C, associated with the loss of adsorbed water. Then, most of the removal of hydration water (4.6% weight loss) takes place between 110 and 250 °C (with an associated sharp endothermic peak centered at around 200 °C), being completed in a softer third step (0.6% weight loss) between 250 and 350 °C. Thus, the overall weight loss corresponding to hydration water is of about 5.2% (7.7% considering also adsorbed water), which corresponds to ca. 0.7 mols of H_2_O per mol of cerium phosphate (CePO_4_·0.7H_2_O, x = 0.7). On the other hand, the DTA-TG curves of C12GA-templated CePO_4_ (Figure 7b) are very similar up to 200–250 °C but, as expected, there is an additional weight loss of ca. 3% between 250 and 430 °C (with an associated wide exothermic band), which corresponds to the decomposition and removal of the dipeptide-based C12GA gelator template. The overall weight loss in this sample was 10.5%. According to these results of thermal analysis, the as-prepared CePO_4_ samples (60 °C-dried) were subjected to subsequent annealing treatments at 250, 600 and 900 °C (air conditions), and the crystal chemical and morphological evolution was investigated (Section 2.3).

### 2.3. Morphology and Crystal-Chemical Characterization of Annealed CePO_4_ Samples (250, 600 and 900 °C)

#### 2.3.1. Crystalline Phase Evolution (XRD) after Annealing Treatments at 250, 600 and 900 °C

The crystalline phase evolution of cerium phosphates upon firing treatment has been already widely investigated. Starting from the hydrated form of hexagonal CePO_4_·xH_2_O (rhabdophane), it normally proceeds with its dehydration (at 110–400 °C) without any change of crystalline system and space group to render anhydrous hexagonal CePO_4_. Some authors state that a complete elimination of crystallization water may not been accomplished up to 600 °C, suggesting also that the presence of water molecules becomes indispensable for the stabilization of hexagonal CePO_4_; thus, the phase transformation into the monoclinic form of CePO_4_ (monazite) would occur after removal of these water molecules [25,101]. This transformation into monoclinic CePO_4_ (monazite) normally occurs in the temperature range between 400 and 700 °C (usually around 600 °C), although formation of monoclinic monazite can be triggered at 200 °C under hydrothermal treatments [5,13,24,39,48,51].

**Figure 8:**
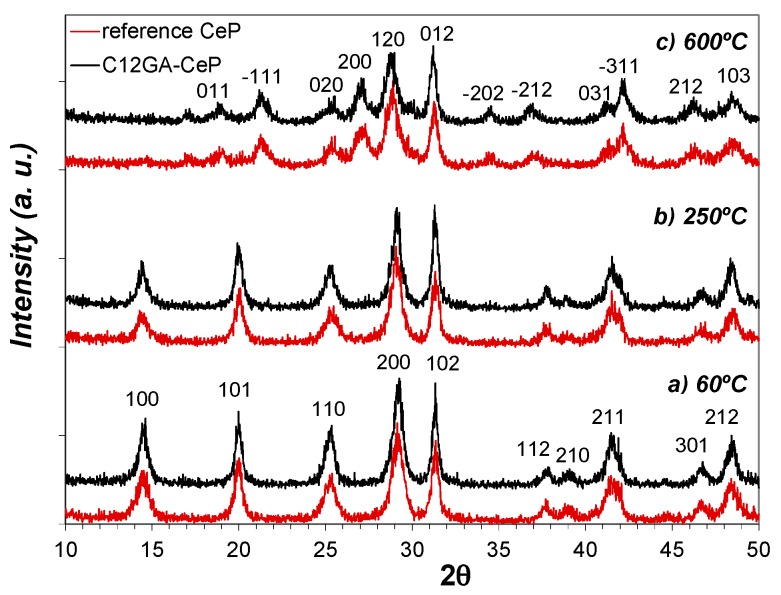
XRD patterns with the evolution of crystalline phase in non-templated reference CePO_4_ (bottom, red-colored) and C12GA-templated CePO_4_ (up; dark-colored): (**a**) as-prepared 60 °C-dried samples; (**b**) 250 °C-annealed samples (air conditions); (**c**) 600 °C-annealed samples (air conditions).

Figure 8 shows the evolution of crystalline phases present in as-prepared reference and C12GA-templated CePO_4_ samples (60 °C-dried) after annealing treatments at 250 and 600 °C (2 h/air atmosphere). The XRD patterns of samples at 60 and at 250 °C are almost identical, and there are also not substantial differences between the patterns of reference and templated CePO_4_ samples. At both temperatures and in both samples, the XRD patterns present relatively broad peaks of small intensity (in accordance with its nanocrystalline character) that correspond to hexagonal rhabdophane, either hydrated at 60 °C (CePO_4_·H_2_O, space group P6_2_22, PDF card # 35-0614) or anhydrous at 250 °C (CePO_4_, space group P6_2_22, PDF card # 75-1880 or also # 34-1380 and # 4-0632). In this respect, it must be noted that the PDF cards of hydrated and anhydrous CePO_4_ are almost identical, being very difficult to differentiate hydrated from anhydrous CePO_4_; the reason may be that some water molecules are always present in hexagonal CePO_4_, as previously commented.

In the case of samples annealed at 600 °C (Figure 8c), the XRD patterns changed in a similar way in both reference and templated CePO_4_, showing the presence of still broader and less intense peaks (very small crystallite size), which mostly corresponds to monoclinic CePO_4_ (monazite, space group P21/n, PDF card # 32-0199). However, the presence of a minor amount of hexagonal CePO_4_ as secondary phase (accompanying the monoclinic monazite) must not be discarded, since the most intense peaks of the hexagonal phase, i.e., the (200) and (102) peaks, overlap with (120) and (012) peaks of monoclinic monazite. This assumption is later corroborated from HRTEM analyses (Section 2.3.2). Thus, XRD patterns indicate that both samples annealed at 600 °C are mainly formed by monoclinic CePO_4_ (monazite) in a very incipient crystallization stadium, and this phase is presumably accompanied by a small amount of still non-transformed hexagonal CePO_4_, in accordance with the phase transformation scheme from hexagonal to monoclinic phase before described, which usually takes place at around 600 °C.

**Figure 9:**
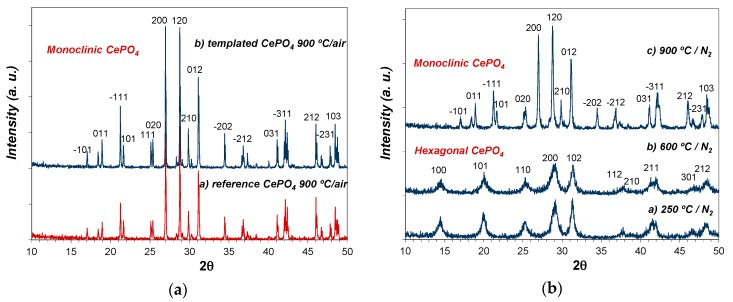
(**a**) XRD patterns of: non-templated reference CePO_4_ (a); and C12GA-templated CePO_4_ (b) after annealing treatment at 900 °C under air atmosphere; (**b**) XRD patterns of templated CePO_4_ after subsequent annealing treatments at: 250 °C (a), 600 °C (b), and 900 °C (c) under N_2_ atmosphere.

As expected, a further annealing treatment at higher temperature (900 °C/2 h; air atmosphere) triggered the transformation and crystallization of monoclinic CePO_4_ (monazite) in both templated and reference samples, showing the corresponding XRD patterns much more intense and narrow diffraction peaks (see Figure 9a). The crystallization of monazite was considerably more advanced (narrower and more intense peaks) in templated CePO_4_ than in reference sample.

As an additional experiment, the pristine C12GA-templated CePO_4_ sample (60 °C) was also subsequently annealed at the same firing temperatures (250, 600 and 900 °C), but this time under N_2_ atmosphere. Noteworthy, in this case the phase transformation from hexagonal to monoclinic CePO_4_ was delayed at higher temperatures (see Figure 9b), having hexagonal CePO_4_ as the only crystalline phase at 600 °C. At this temperature, the crystallization degree of the hexagonal phase is also very poor (small crystallite size according to the observed broad and weak-intensity peaks). Given that in this case the phase transformation must then occur above 600 °C, monazite crystallization at 900 °C is also much less advanced, presenting broader and less intense diffraction peaks with respect to the sample annealed under air conditions.

#### 2.3.2. Morphology and Crystal-Chemical Evolution (FE-SEM, TEM and HRTEM)

**Figure 10:**
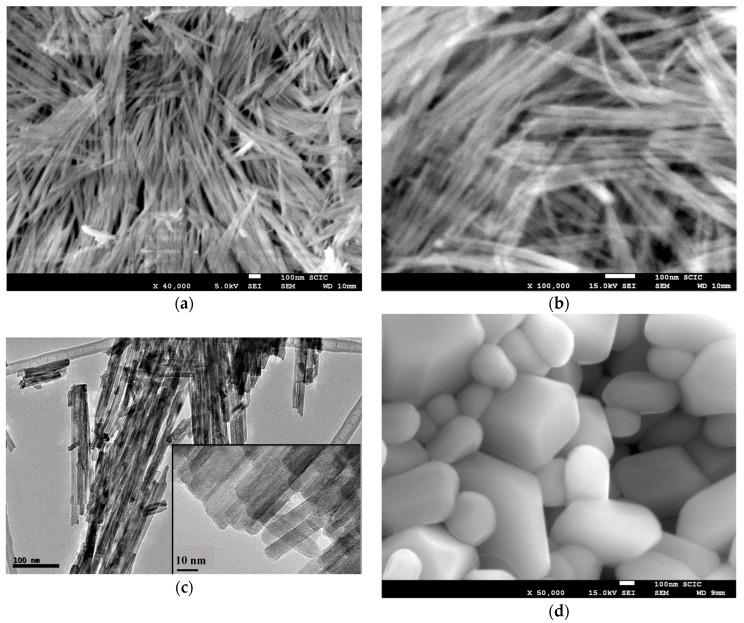
FE-SEM images (**a**,**b**) and TEM images (**c**) of templated nanofibrous CePO_4_ annealed at 600 °C (air conditions); (**d**) FE-SEM image of templated CePO_4_ annealed at 900 °C (air conditions).

The morphology of C12GA-templated CePO_4_ sample once annealed at 600 °C (2 h/air conditions) is shown in the FE-SEM and TEM images of Figure 10a–c. Although most of hexagonal CePO_4_ has been already transformed into nanocrystalline monoclinic monazite at this temperature (according to XRD results), the templated CePO_4_ sample still conserves the same homogeneous nanofibrous morphology (Figure 10a,b) of as-prepared 60 °C-dried xerogel, consisting of very thin, elongated and poorly entangled nanofibrils around 20 nm thick and 1 micron length, which are forming a mesh of fibrous bundles. The higher magnification TEM images (Figure 10c) reveal again a morphology based on rather straight and rod-like nanofibrils (ca. 10 nm thick and hundreds of nanometers length), merged into slightly entangled sheet-like nanotapes. In contrast, the morphology of the reference (non-templated) CePO_4_ sample annealed at 600 °C (Figure S3) contained amorphous regions with more rounded shapes and with shorter (lower aspect ratio) and entangled nanofibers forming rather bulk aggregates.

A more precise crystal-chemical characterization of some regions of templated-CePO_4_ sample annealed at 600 °C (containing well-detached individual or not juxtaposed nanofibrils) was also performed by high-resolution transmission electron microscopy (HRTEM), and confirmed the presence of relatively abundant hexagonal CePO_4_ nanofibers (see Figure S4), as it was previously suggested in the XRD discussion.

However, the nanofibrous morphology collapsed after the subsequent annealing treatment at 900 °C (for 2 h, and under air atmosphere), owing to the extensive crystallization and growing of monoclinic CePO_4_ (monazite). FE-SEM images of both templated (Figure 10d) and reference CePO_4_ samples (Figure S5) annealed at 900 °C under air atmosphere show the absence of nanofibers and, instead, both samples consist of micron-sized aggregates, with very small nanoparticles (200–500 nm sized) and well-defined and shaped rounded morphologies (slightly more prismatic in the case of C12GA-templated sample.

**Figure 11:**
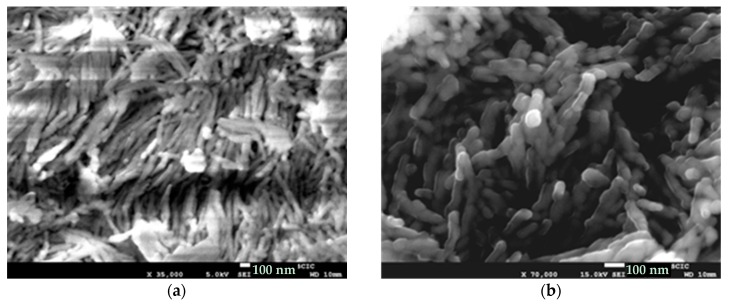
FE-SEM images corresponding to C12GA-templated nanofibrous CePO_4_ annealed at 900 °C under N_2_ conditions: (**a**) Well-preserved nanofibrous region (x 35000); (**b**) Higher magnification region (x 70000) showing thicker, more grown and aggregated nanofibers. Bar length: 100 nm.

Interestingly, the C12GA-templated CePO_4_ sample preserved to a high extent its nanofibrous morphology at 900 °C when the annealing treatments were performed under N_2_ atmosphere (Figure 11). As it was discussed previously (Section 2.3.1), in this case the transformation into monoclinic monazite occurs above 600 °C and the crystallization degree of this phase at 900 °C is not so advanced as when the annealing is performed under air conditions. The FE-SEM images of Figure 11 show some regions of this templated sample (900 °C/N_2_), in which the nanofibrous regions may be clearly appreciated (Figure 11a). At this temperature, these nanofibrils appear to be thicker or more grown (around 50 nm thick) and aggregated, having also more rounded morphologies (Figure 11b). This morphology would correspond to an incipient stadium of crystalline growth, which would lead (with a further increase of annealing time or temperature) to the grain-like rounded nanoparticles previously observed for the sample annealed under air conditions (Figure 10d). 

**Figure 12:**
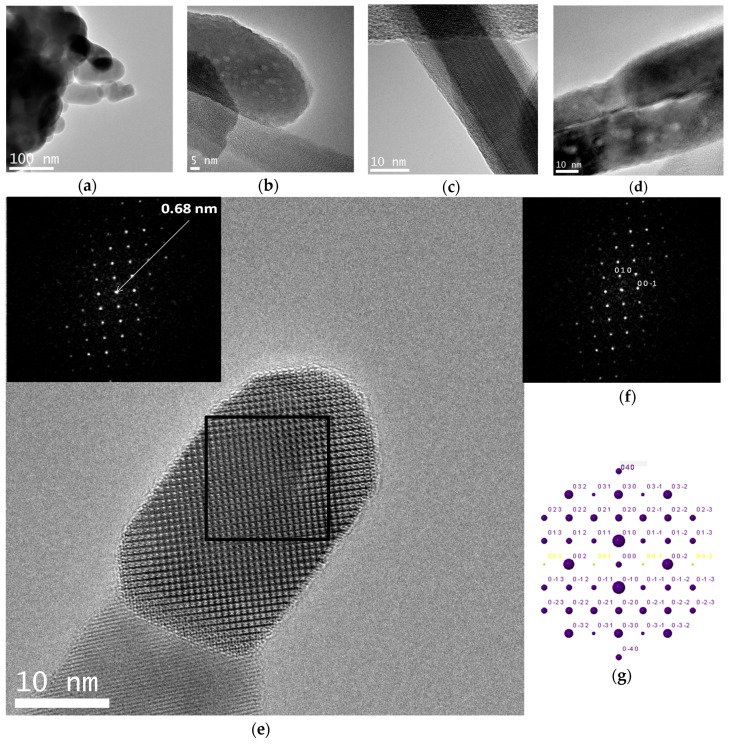
(**a**–**e**) HRTEM images of a C12GA-templated CePO_4_ annealed at 900 °C under N_2_ conditions, the inset of (**e**) showing the corresponding digital diffraction pattern (DDP) of the region of the nanofiber marked with an square, with indicated distance; (**f**) same DDP with some indexed (*hkl*) values; (**g**) corresponding simulated kinetic diffraction diagram.

A closer inspection and crystal-chemical characterization of this sample was also performed by HRTEM (Figure 12). The HRTEM images of Figure 12a,b confirm the morphology change experienced with respect to 600 °C-annealed samples, since the nanofibers have transformed into regions of greater thickness and smaller size or aspect ratio. However, nanofibrous regions with thinner nanofibrils are still preserved (see e.g., Figure 12c,d). The crystalline lattice fringes of a representative nanofiber (10–15 nm thick) of this 900 °C-annealed sample (under N_2_ atmosphere) may be clearly appreciated in the HRTEM image of Figure 12e, and the corresponding digital diffraction pattern (DDP) of the square-marked region (taken along the [100] zone axis) shows diffraction spots associated to inter-planar distances (0.65 and 0.68 nm) and angles (90) that can only be assigned to monoclinic CePO_4_. The DDP of Figure 12f shows the assignment of some of these spots to the corresponding (hkl) lattice planes of the monoclinic system, and the corresponding simulated kinetic diffraction diagram is also shown in Figure 12g.

### 2.4. Elucidation of Ce Oxidation State (Ce^3+^ vs. Ce^4+^) in CePO_4_ Samples by STEM-EELS Spectroscopy

In order to get more detailed information about the chemical composition, i.e., the oxidation state of cerium ions (Ce^3+^ vs. Ce^4+^) in the obtained CePO_4_ materials, an electron energy loss spectroscopy (EELS) study was also carried out. In this respect, the elucidation of the presence of some amount of Ce^4+^ along with Ce^3+^ ions (the Ce^4+^/Ce^3+^ ratio) becomes especially important, since it may strongly affect the photo-luminescence properties of the obtained CePO_4_ materials. In this respect, although the coexistence of Ce^4+^ ions becomes generally detrimental for the luminescent emission because of its competitive absorption in the UV region, in some systems the presence of Ce^4+^ along with Ce^3+^ has induced and interesting an strong blue emission [4].

**Figure 13:**
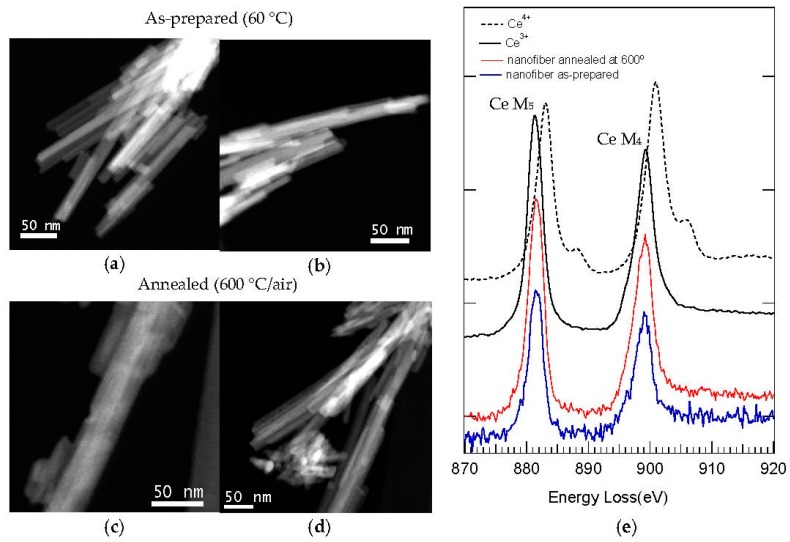
(**a**–**d**) STEM-HAADF images of C12GA-templated CePO_4_ sample, as-prepared (**a** and **b**) and annealed at 600 °C/air (**c** and **d**); (**e**) Corresponding EELS spectra performed in a nanofiber of as-prepared and 600 °C-annealed templated CePO_4_; For comparison purposes, the electron energy loss near edge structure (ELNES) spectra of Ce^3+^ and Ce^4+^ ions are also shown as a reference [102].

With this aim, EELS spectra of as-prepared (60 °C) and 600 °C-annealed samples (Figure 13e) were recorded from STEM-HAADF electron microscopy images (Figure 13a–d), and most attention was given to the electron energy loss in the near edge structure (ELNES) of Ce, i.e., the M_4_ and M_5_ edge. This M_4_,_5_ edge exhibits in Ce distinct valence-specific shapes slightly separated in energy, being best suited to discriminate the presence of Ce^3+^ or Ce^4+^ ions [4,103]. As it is shown in the reference ELNES spectra for both ions also included in Figure 13e [102], the M_4_ peak (901.0 eV) is more intense than the M_5_ peak (883.1 eV) in Ce^4+^, while the contrary occurs in Ce^3+^ (M_5_ peak at 881.4 eV is more intense than M_4_ peak at 899.4 eV). Moreover, the EELS spectrum of Ce^4+^ also presents as characteristic signal a small shoulder after both peaks.

The EELS spectra (Figure 13e) recorded from representative nanofibers of templated CePO_4_ samples, 60 °C-dried and 600 °C-annealed (Figure 13a–d), show in both cases that the Ce M_5_ peak is the most intense (indicative of Ce^3+^). Considering also the position of the peaks, its symmetric shape and the absence of the characteristic signal of Ce^4+^ ions (a shoulder after both peaks), it can be concluded that the prepared CePO_4_ materials contain exclusively Ce^3+^ ions. The as-prepared reference sample exhibited also a similar EELS spectrum, indicating the presence of Ce^3+^ (Figure S6).

### 2.5. Optical Absorption and Photo-Luminescence Properties of CePO_4_ Samples

**Figure 14:**
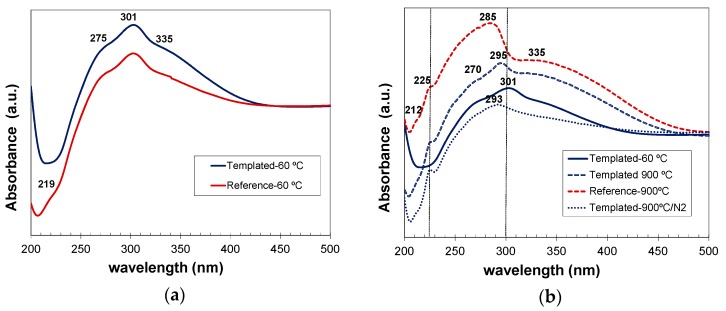
Absorbance spectra of: (**a**) reference and C12GA-templated as-prepared CePO_4_ samples (60 °C-dried); and (**b**) reference and templated CePO_4_ materials after annealing at 900 °C (under air or N_2_ conditions), showing also the spectrum of 60 °C-dried templated sample for comparison purposes.

Finally, the photoluminescence (PL) properties of non-templated and C12GA-templated CePO_4_ materials were evaluated (in both as-prepared and annealed samples). Previously, the optical absorption spectra of as-prepared and annealed samples were obtained (Figure 14), enabling also to select the optimal excitation wavelengths for the PL experiments.

As-prepared reference and C12GA-tempated samples based on hexagonal CePO_4_ rhabdophane exhibited very similar absorption spectra (Figure 14a), mainly concentrated in the UV region, with a broad band extending from 230 to almost 420 nm. This UV absorption was slightly more intense in the case of templated sample (which contained more uniform and elongated nanofibers). The major absorption peak is observed at around 301 nm coupled with a shoulder at 275 nm and another one at around 335–350 nm with the cutoff wavelength at ca. 422 nm. These absorption features are in good agreement with the typical transitions of Ce^3+^ from its 2F (4f^1^) ground state to the five crystal field split levels of 2D (5d^1^) excited states [4,20,48], although the absorptions are slightly red-shifted (around 20 nm) with respect to previous reports [4,48]. 

In the case of samples annealed at 900 °C under air conditions and containing well-grown (non-fibrous) monoclinic CePO_4_, the optical absorption was considerably enhanced (with respect to as-prepared samples) and also slightly blue-shifted (Figure 14b). In these samples, a broad band with several small shoulders extends from 200 nm up to the major absorption peak, which appears now at around 285–295 nm, and then a less intense and broad absorption shoulder is also observed from 310 to almost 475 nm. These features fit also very nicely with the 4f to 5d transitions of Ce^3+^, which presents its characteristic (most intense) peaks between 200–300 nm [4]. Noteworthy, the spectra of templated samples annealed at 900 °C under air (non-fibrous), and specially the sample annealed under N_2_ conditions (still conserving the nanofibrous morphology), exhibited absorption features more similar to those of as-prepared (60 °C-dried) nanofibrous CePO_4_ sample.

Considering the above-described absorption behaviour, the photoluminescence (PL) spectra were recorded at slightly different excitation wavelengths for as-prepared (301 nm) and for annealed samples (275 nm). As it is well-known, CePO_4_ presents a typical PL emission in the UV region, which is associated to 5d to 4f transitions of Ce^3+^ ions. Moreover, CePO_4_ 1D-nanostructures (nanofibers, nanorods,...) may exhibit in some cases enhanced PL properties with respect to its conventional bulk counterparts, and these properties depend also very much on the morphology and crystallinity, which must be optimized for a better performance in optoelectronic devices [1,5,13,52,56].

**Figure 15:**
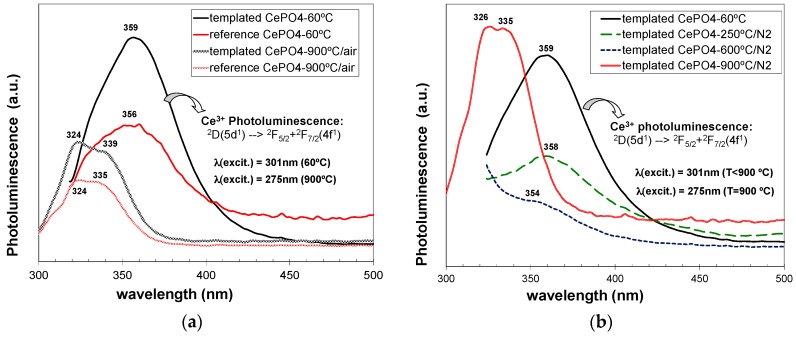
Photoluminiscence emission spectra corresponding to: (**a**) non-templated reference and C12GA-templated CePO_4_ samples, as-prepared (60 °C) and after annealing at 900 °C/air; and (**b**) comparison of photoluminescence emission of as-prepared (60 °C) templated CePO_4_ with respect to corresponding CePO_4_ once annealed at 250, 600 and 900 °C (under N_2_ atmosphere).

C12GA-templated as-prepared CePO_4_ exhibited (Figure 15a) a relatively intense PL emission mostly confined in the UV region, with a broad emission band extending between 320 and 430 nm. This emission band must be associated with the transition decay from the ^2^D(5d^1^) state to the two split 4f^1^ ground states of ^2^F(4f^1^) (^2^F_5/2_ + ^2^F_5/2_) [4]. In addition, the possible blue-emission (around 400–550 nm) associated with a charge transfer between Ce^4+^ donation centers and Ce^3+^ luminescence centers was not observed [4], also confirming the absence of Ce^4+^ ions in as-prepared CePO_4_. Very noteworthy, the photoluminescence emission of templated CePO_4_ was considerably enhanced with respect to non-templated reference sample. This fact could be attributed to the improved morphology and crystallinity characteristics of the sample prepared through the use of the C12GA template as nanofibrous scaffold [1,52]. This sample contained higher aspect ratio nanofibers, with a more uniform morphology and narrow-size distribution, and also with an improved crystallinity (better-defined facets and single crystalline features), therefore confirming the success of the employed templating strategy.

However, both reference and C12GA-templated samples annealed at 600 °C did not exhibit luminescence properties. This PL extinction has been already associated in previous reports with the much-less homogeneous character and poor crystallinity of CePO_4_ materials at this temperature range (400–700 °C), as a consequence of the phase transformation from hexagonal to monoclinic CePO_4_. In our case, CePO_4_ samples annealed at 600 °C contained both hexagonal and monoclinic CePO_4_ phases, with considerably lower crystallinity and morphology uniformity, and this could lead to the occurring photoluminescence extinction at this temperature [13].

With a further annealing at 900 °C (under air conditions), the PL emission was recovered again in both reference and templated materials (Figure 15a), associated with the complete transformation and advanced crystallization degree of monoclinic CePO_4_ (monazite). This PL emission at 900 °C was strongly blue-shifted (appearing the band at 300–360 nm) with respect to the emission observed in hexagonal-based (60 °C-dried) CePO_4_ materials (320–430 nm). As in the case of as-prepared samples, templated CePO_4_ showed a considerably higher emission with respect to reference sample. It must be also highlighted that, although monoclinic CePO_4_ (monazite) is supposed to have improved photoluminescence emission than hexagonal CePO_4_, due to the luminescence quenching effect induced by the water molecules still retained in hexagonal CePO_4_ [22,104], in our case the emission of both monazite-based CePO_4_ samples (900 °C-annealed in air conditions) was considerably lower than the emission shown by as-prepared (hexagonal) CePO_4_. Again, this fact could be explained by the presence of uniform nanofibrous morphologies in as-prepared CePO_4_ materials, in contrast with the rounded nano-grained bulk aggregates with non-uniform morphologies present in both monazite-based CePO_4_ samples annealed at 900 °C under air conditions. Indeed, C12GA-templated sample annealed at 900 °C under N_2_ conditions, which conserved to a high extent the nanofibrous morphology (and with a less-advanced monazite crystallization), exhibited a higher luminescence than as-prepared hexagonal-based CePO_4_ (see Figure 15b). The decrease of luminescence of C12GA-templated sample with increasing the annealing temperature at 250 and then at 600 °C under N_2_ conditions may be also appreciated in Figure 15b, which would be attributed, as previously explained, to the reduced crystallinity (amorphization) and increased heterogeneity caused by the hexagonal to monoclinic phase transformation.

## 3. Conclusions

In this investigation, a novel and relatively simple transcription strategy has been designed for the template-synthesis of cerium(III) phosphate nanofibers having an improved nanofibrous morphology using a pH-sensitive nanofibrous hydrogel (based on a glycine-alanine lipodipeptide gelator, C12GA) as structure-directing scaffold.

With this aim, a phosphorylated hydrogel nanofibrous template was first prepared by soft acidification (pH ca. 4.5) of a C12GA basic solution with H_3_PO_4_, which exhibited a morphology based on long nanoribbons or nanotapes (60–100 nm width, 100–140 nm thick and hundreds of microns length) merged laterally forming broader tapes (up to 3–5 microns width) and a relatively entangled 3D network. This phosphorylated hydrogel presented a crystalline character associated to NaH_2_PO_4_ crystallization and also to the periodic self-assembly of C12GA gelator molecules into a layered structure, with a preferential 2D-growth leading to nanotapes formation. The energy-minimized model proposed to explain this aggregation behavior, in accordance with results of FTIR and WAXD measurements, indicated that the self-assembly was mainly driven by hydrophobic effects, along with a small contribution of H-bonding.

This nanofibrous hydrogel was then employed as a template to direct the mineralization of high aspect ratio nanofibrous cerium phosphate (hydrated and anhydrous forms), which in situ-formed by post-diffusion (infiltration) of aqueous CeCl_3_ and subsequent drying (60 °C) and annealing treatments (at 250, 600 and 900 °C). Noteworthy, this templating approach successfully led to the formation of hydrated cerium(III) phosphate nanofibrous bundles presenting rather co-aligned and elongated nanofibers (10–20 nm thick and up to ca. 1 μm long). The formed nanofibers consisted of hexagonal (P6_2_22 space group) CePO_4_·xH_2_O rhabdophane nanocrystals at 60 and 250 °C (x = 0.7 at 60 °C according to DTA/TG analysis), as it was confirmed by XRD and HRTEM characterization, with a much better-grown and more homogeneous fibrous morphology with respect to a reference CePO_4_ sample prepared under similar (non-templated) conditions.

Interestingly, the nanofibrous morphology was completely preserved after annealing treatment at 600 °C/air, although at this temperature the sample was already mostly constituted by poorly crystallized monoclinic CePO_4_ arising from the still incomplete phase transformation from hexagonal to monoclinic CePO_4_ (monazite, P21/n space group). A further annealing treatment under air conditions caused the complete transformation into monoclinic monazite, with an advanced crystallization degree, but also accompanied by the collapse of the nanofibrous morphology to give well-grown aggregates formed by nano-grained particles. However, and very remarkably, the nanofibrous morphology was highly preserved by performing the subsequent annealing treatments up to 900 °C under N_2_ atmosphere.

STEM-EELS analyses performed with as-prepared and 600 °C-annealed samples, paying especial attention to the M_4_,_5_ edge structure (ELNES) of Ce, confirmed the exclusive presence of Ce^3+^ in the obtained materials, discarding the coexistence of Ce^4+^ ions. Remarkably, the nanofibrous as-prepared (60 °C-dried) hexagonal CePO_4_ exhibited an enhanced UV photoluminescence emission (associated to 5d to 4f transitions of Ce^3+^ ions) with respect to the reference sample prepared under similar (non-templated conditions), and this was associated to the higher aspect ratio, more uniform morphology and improved crystallinity (better-defined facets and single crystalline features) of the nanofibers forming the C12GA-templated CePO_4_ sample. The incomplete phase transformation into monoclinic monazite after annealing at 600 °C, resulting in a heterogeneous microstructure of the samples containing both monoclinic and hexagonal phases, induced the extinction of photoluminescence emission at this temperature, which was partially recovered and slightly blue-shifted after complete transformation and development of well-grown monazite nano-grained aggregates. Also noteworthy, the monoclinic CePO_4_ sample annealed at 900 °C under N_2_ conditions, and still preserving the nanofibrous morphology, showed an enhanced photoluminescence emission with respect to as-prepared nanofibrous hexagonal rhabdophane.

## 4. Materials and Methods

### 4.1. Preparation of C12GA Lipodipeptide Gelator and of HCl-C12GA and H_3_PO_4_-C12GA Hydrogels/Xerogels

C12GA hydrogelator compound shown in Scheme 1 (*N*-Dodecanoyl-glycyl-l-alanine, formula weight: 328.24 g/mol) was prepared as previously described (e.g., gelator *3* in [77] or *3c* in [82]), following a Schotten-Baumann procedure. With this pH-sensitive (or pH-responsive) C12GA gelator compound two hydrogels were obtained by pH change (acidification):

(i) In the case of *HCl-C12GA hydrogel*, 5 mg of C12GA gelator (1.5 × 10^−5^ mol) were dissolved in 1 mL of 0.1 M aqueous NaOH under heating conditions (thus having a 15 mM C12GA concentration and a NaOH:C12GA molar ratio of 6.7:1), and hydrogelation of the transparent hot solution was induced by free-cooling to room temperature and soft acidification by exposure to HCl vapors in a sealed glass chamber. A dried xerogel of the formed hydrogel was obtained by liophilization to be characterized by SEM/TEM electron microscopies and FTIR spectroscopy.

(ii) For the preparation of the phosphorylated *H_3_PO_4_-C12GA hydrogel* (used as nanofibrous template for the transcription of nanofibrous CePO_4_), 50 mg of C12GA gelator (1.5 × 10^−4^ mol) were dissolved in 2.5 mL of 2.5 M aqueous NaOH (5 × 10^−3^ mol) in a screw-capped glass vial under heating and stirring conditions. Then, in order to induce the formation of a phosphorylated hydrogel, the transparent solution was acidified by instantaneous addition of 5.5 mL of 1 M aqueous H_3_PO_4_ (5.5 × 10^−3^ mol), thus having a final pH of ca. 4, a C12GA concentration of 19 mM and NaOH:C12GA and H_3_PO_4_:NaOH molar ratios of 33.3:1, and 1.1:1, respectively. After a few-seconds homogenization under heating conditions and vigorous stirring, the reaction vial containing the transparent solution was placed in an ice-bath inducing both rapid cooling and instantaneous hydrogelation. The as-prepared hydrogel was left drying (opened vial) at open air during 1 h and then employed as nanofibrous template for the mineralization of nanofibrous CePO_4_ (Section 4.2 below). A replica of H_3_PO_4_-C12GA hydrogel was dried by liophilization (similarly to HCl-C12GA hydrogel) and characterized by SEM/TEM and FTIR techniques.

### 4.2. Transcription of Nanofibrous CePO_4_·xH_2_O Using the H_3_PO_4_-C12GA Hydrogel as Nanofibrous Template

The as-prepared phosphorylated H_3_PO_4_-C12GA hydrogel (after 1 h-drying) was then employed as nanofibrous scaffold to direct the mineralization of nanofibrous CePO_4_. With this aim, 3 mL of an aqueous solution of 1.67 M CeCl_3_·7H_2_O (5 × 10^−3^ mol of Ce^3+^, thus having a H_3_PO_4_:Ce^3+^ molar ratio of 1.1:1) was softly added dropwise at the top part of the previously formed H_3_PO_4_-C12GA hydrogel (see the illustrating Scheme 2 in Section 2.2.1), and this upper layer of Ce^3+^ solution was left to spontaneously impregnate (post-diffusion strategy) the subjacent pre-formed layer of H_3_PO_4_-C12GA nanofibrous hydrogel during 48 h (closed vial). The impregnation (infiltration) of the Ce^3+^ solution through the pores of the pre-formed phosphorylated hydrogel took place easily and without disruption of the hydrogel state (dissolution of the gel did not occur), giving rise to a homogenous single layer of CeCl_3_-impregnated H_3_PO_4_-C12GA hybrid hydrogel. The obtained hybrid C12GA-NaH_2_PO_4_-CeCl_3_ hydrogel was then softly dried in an oven (60 °C/air/72 h) to trigger the mineralization of nanofibrous CePO_4_ templated by the nanofibrous C12GA-NaH_2_PO_4_ scaffold (by double-exchange or metathesis reaction between CeCl_3_ and NaH_2_PO_4_ species grafted or entrapped within the nanofibrous hydrogel, giving rise to CePO_4_ and NaCl), and finally it was thoroughly washed with water until complete elimination of NaCl. The resulting hybrid C12GA-CePO_4_ xerogel, either as-prepared (60 °C-dried) or after subsequent annealing treatments in electrical furnace at 250, 600 and 900 °C (under air or N_2_ atmosphere, with a heating speed of 2 °C/min and a soaking time of 2 h) was then submitted to characterization (see Section 4.4).

### 4.3. Preparation of a Non-Templated Reference Sample of CePO_4_·xH_2_O (Reference-CePO_4_)

A reference, non-templated CePO_4_ sample was prepared under synthesis conditions as similar as possible to those employed with C12GA-templated CePO_4_ sample. With this aim, 5.5 mL of 1 M aqueous H_3_PO_4_ (5.5 × 10^−3^ mol) were rapidly added to a hot solution of 2.5 mL of aqueous 2.5 M NaOH (5 × 10^−3^ mol) and, after a few-seconds homogenization under heating and stirring conditions, the resulting NaH_2_PO_4_-based solution was rapidly cooled in an ice bath, and then left at open air (opened glass vial) during 1 h. After this room-temperature conditioning, 3 mL of an aqueous solution of 1.67 M CeCl_3_·7H_2_O (5 × 10^−3^ mol of Ce^3+^, thus having a H_3_PO_4_:Ce^3+^ molar ratio of 1.1:1) were added dropwise under stirring conditions to the former NaH_2_PO_4_-based solution (immediate precipitation of a white powder of CePO_4_·xH_2_O occurred), and left ageing for 48 h (closed vial) before being submitted to subsequent drying (opened vial) at 60 °C (air/72 h) and annealing treatments at 250, 600 and 900 °C, similarly as with C12GA-templated Ce_3_PO_4_ sample.

### 4.4. Characterization of C12GA Xerogels and of as-Prepared (60 °C) and Annealed CePO_4_ Samples

Dried C12GA xerogels, reference and hybrid C12GA-CePO_4_ as-prepared samples (60 °C) and the resulting annealed CePO_4_ powders (250, 600 and 900 °C) were characterized by the following solid-state characterization techniques:

#### 4.4.1. Thermal Analysis

Simultaneous differential thermal and thermogravimetric analysis (DTA/TG) of as-prepared reference CePO_4_ and C12GA-CePO_4_ dried samples (60 °C) was carried out with a Mettler Toledo thermal analyzer (under air conditions, using Pt crucibles and with a constant 10 °C/min heating from 25 up to 1000 °C, Columbus, OH, USA).

#### 4.4.2. X-ray Powder Diffraction (WA)XRD

Wide-angle X-ray powder diffraction (WAXD) characterization of HCl-C12GA and H_3_PO_4_-C12GA xerogels was performed at room temperature with a Bruker D4 Endeavor X-ray powder diffractometer (Billerica, MA, USA) by using Cu-K_α_ radiation. Samples of the powdered xerogels were placed on a sample holder and data were collected for 2θ values between 2 and 40° with a step size of 0.03° and a time step of 10 s. On the other hand, conventional X-ray powder diffraction (XRD) patterns of as-prepared and annealed CePO_4_ powdered samples were recorded at room temperature in a Siemens D-500 powder diffractometer (Munich, Germany) with CuK_α_ radiation. In this case, the data were collected for 2θ values between 10° and 50° with a step size of 0.02° and a time step of 2 s.

#### 4.4.3. Fourier-Transformed Infrarred (FTIR) Spectroscopy

FTIR spectra of powdered dried (lyophilized) HCl-C12GA and H_3_PO_4_-C12GA xerogels were recorded with a JASCO-FTIR-6200 spectrometer (Oklahoma City, OK, USA). For the measurements, KBr pellets of the xerogels were made by mixing the xerogel powder (1–2 mg) and dry KBr (200 mg) in an agate mortar and pressing the mixture at 10 ton for ca. 10 min (obtaining discs of less than 0.5 mm).

#### 4.4.4. Electron Microscopy Characterization (FE-SEM, TEM, HRTEM and STEM-HAADF)

The morphology, crystalline structure and chemical composition of CePO_4_ nanofibers were characterized by different electron microscopy techniques. Field-Emission Scanning Electron Microscopy observations (FE-SEM) were performed with a JEOL 7001F electron microscope (Tokyo, Japan), following conventional preparation and imaging techniques. The chemical composition and homogeneity of as-prepared (60 °C) samples were also determined by semi-quantitative elemental analysis with an EDX analyzer (supplied by Oxford University) attached to this microscope. Further microstructure details of the obtained rod-like CePO_4_ nanofibers were obtained by Transmission Electron Microscopy (TEM) in a JEOL JEM-1010 (100 kV) microscope. For these TEM observations, the micronized powders were suspended in water and deposited after sonication onto conventional holey carbon grids.

Additional and more precise information of the morphology and crystalline structure of the formed CePO_4_ nanofibers was obtained from High-Resolution Transmission Electron Microscopy (HRTEM) images and corresponding digital diffraction patterns (DDPs), and also from Scanning Transmission Electron Microscopy working in High Angle Annular Dark-Field (STEM-HAADF) images obtained in a JEOL 2010F microscope equipped with an HAADF detector.

#### 4.4.5. Determination of Ce^4+^/Ce^3+^ Ratio from STEM-EELS Spectroscopy

The oxidation state of Ce ions (Ce^3+^ vs. Ce^4+^) in CePO_4_ powders was determined by Electron Energy Loss Spectroscopy (EELS) in the same JEOL 2010F microscope equipped with an EELS analyzer. All EELS spectra were recorded in STEM-HAADF mode using energy dispersion of 0.3 eV/channel and acquisition times of 0.3–10 s. For the determination of the M_5_/(M_4_ + M_5_) [103] and M_4_/M_5_ [4] intensity ratios, the recorded EELS spectra were adjusted by Gaussian curves using an specific software.

#### 4.4.6. Optical Absorption and Photo-Luminescence Spectra

Photoluminescence properties of powdered samples (reference and templated CePO_4_ materials) were measured at room temperature using a Nd-YAG pulsed laser (Vibrant System 355 II, Opotek, Carlsbad, CA, USA) and recording the spectra with a Black-Comet-SR (Stellarnet, Tampa, FL, USA) spectrometer. Previously, the UV-vis absorption spectra of samples were recorded with a Jasco V670 spectrophotometer (by diffuse reflectance technique).

## Supplementary Materials

The following are available online at www.mdpi.com/2310-2861/3/2/23/s1.
