# Supplementary material for: Transcription of Nanofibrous Cerium Phosphate Using a pH-Sensitive Lipodipeptide Hydrogel Template
Source: Gels. 2017 Jun 10;3(2):23. doi: 10.3390/gels3020023 (PMC6318699; doi:10.3390/gels3020023)
Supplement: Supplementary file 1 [file gels-03-00023-s001.pdf]

gels

MDPI

Supplementary Materials for:

Transcription of Nanofibrous Cerium Phosphate Using a pH-Sensitive Lipodipeptide Hydrogel Template

Mario Llusar, Beatriu Escuder, Juan de Dios López-Castro, Susana Trasobares and Guillermo Monrós

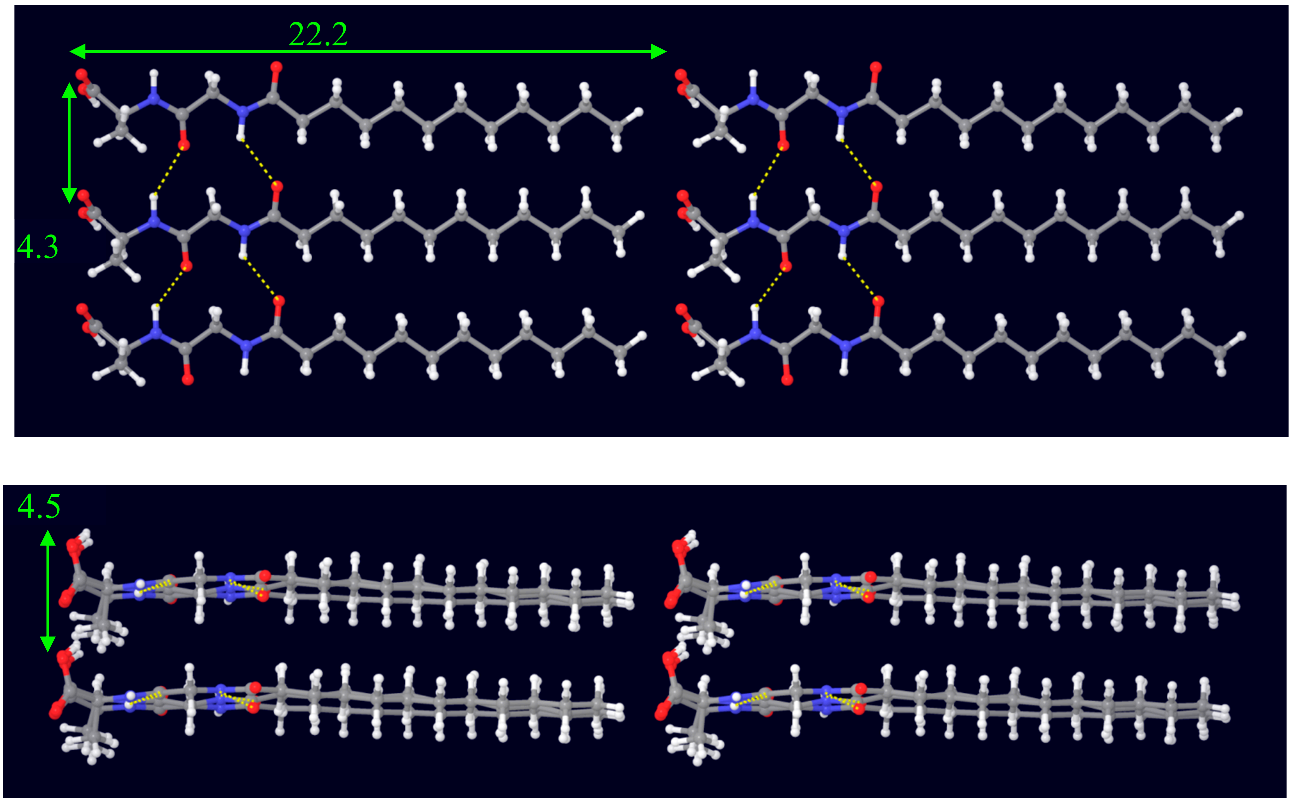

Figure S1. Alternative Energy-minimized structure models (MACROMODEL 7.0, AMBER*) [98] for the packing of compound ***C12GA*** in phosphorylated xerogel (non-polar hydrogens are omitted for clarity in the bottom image).

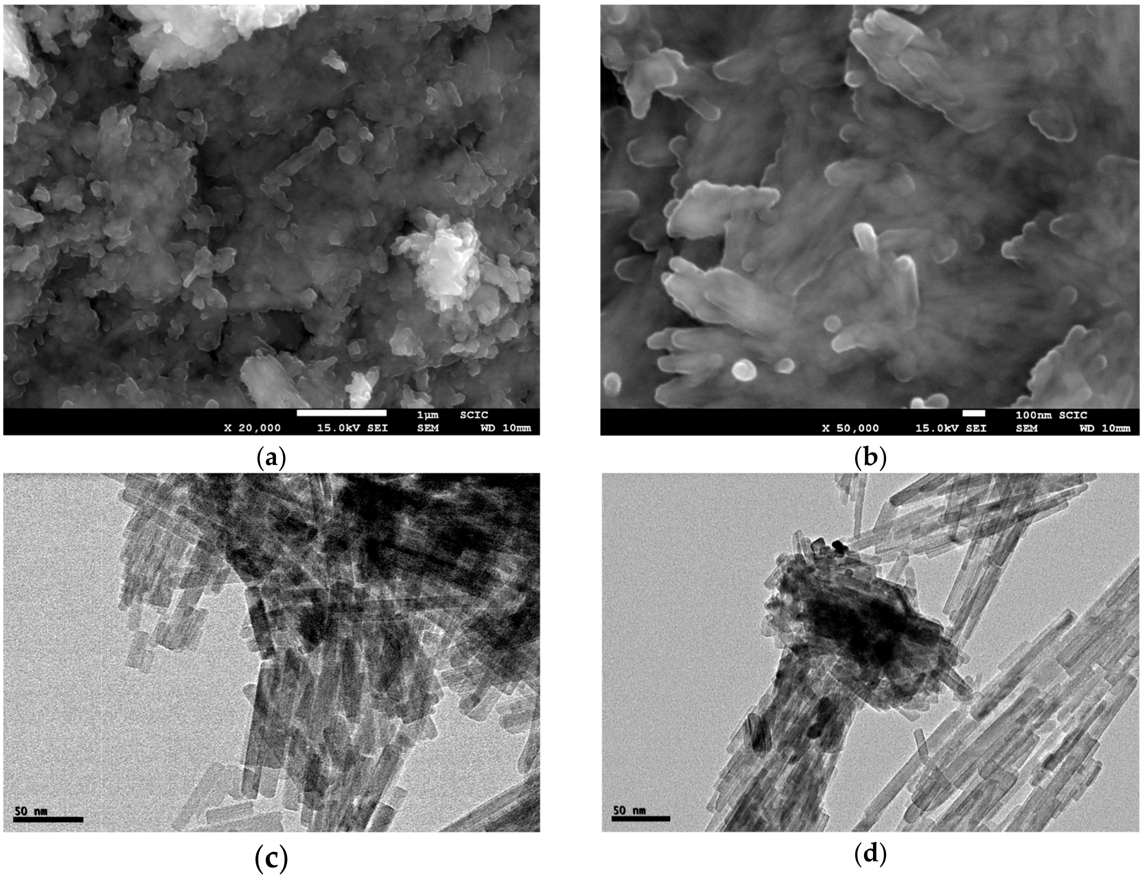

Figure S2. FE-SEM images (**a**,**b**); and TEM images (**c**,**d**) of as-prepared reference $CePO_4$ (non-templated, 60 °C-dried and washed sample).

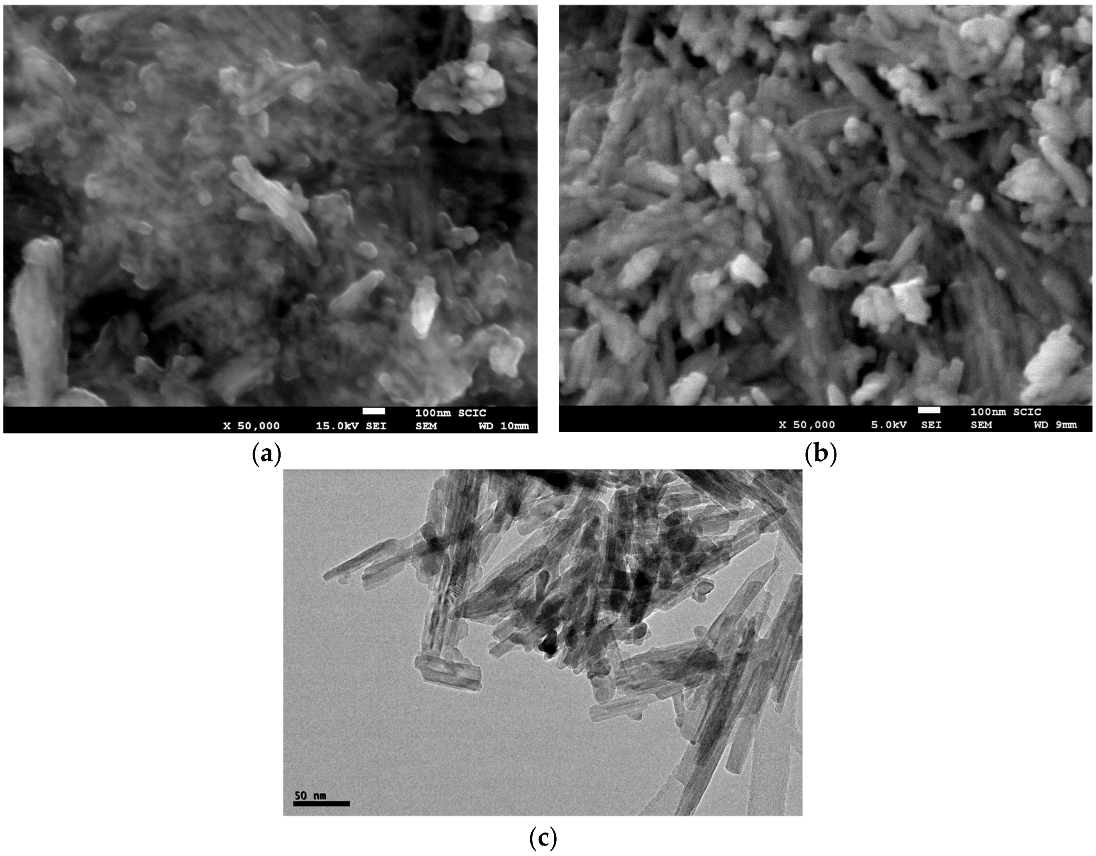

Figure S3. FE-SEM (**a**,**b**); and TEM images (**c**) of non-templated reference $CePO_4$ annealed at 600 °C.

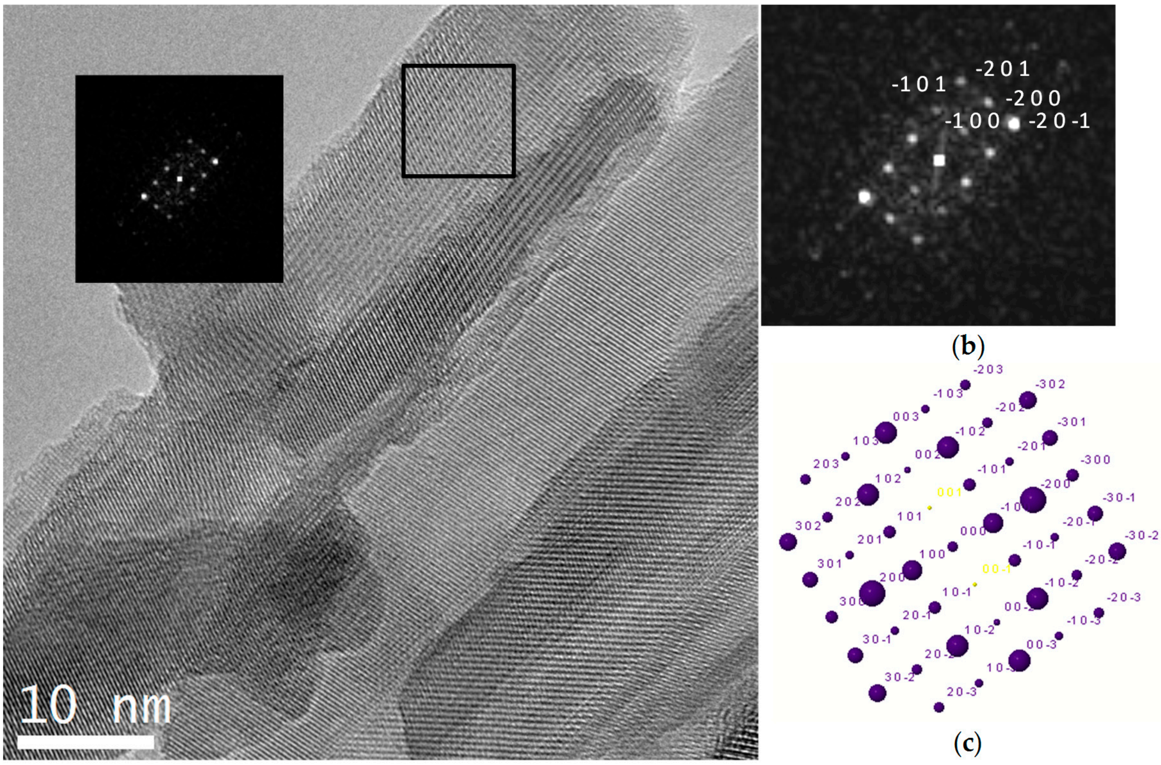

(**a**) (**b**) (**c**)

Figure S4. (**a**) HREM image of representative nanofibers of C12GA-templated nanofibrous $CePO_4$ annealed at 600 °C under air conditions, the inset showing the corresponding digital diffraction pattern (DDP) of the region of the nanofiber marked with an square; (**b**) the same DDP taken along the [010] zone axis, indicating some indexed (*hkl*) facets, which must be unambiguously assigned to hexagonal $CePO_4$; and (**c**) the corresponding simulated kinetic diffraction diagram.

The HRTEM image of Figure S4 shows some of these nanofibrils (around 10-15 nm thick), and the corresponding digital diffraction pattern (DDP) presents diffraction spots associated to inter-planar distances and angles (d1/d2/angle: 0.46/0.28/20.0, 0.46/0.31/43.4 and 0.46/0.61/42.8) that can only be assigned to hexagonal $CePO_4$. The DDP of Figure S4b shows the assignment of some of these spots to the corresponding (hkl) lattice planes of the hexagonal system ([uvw] zone axis: [010]), and the corresponding simulated kinetic diffraction diagram is also shown in Figure S4c.

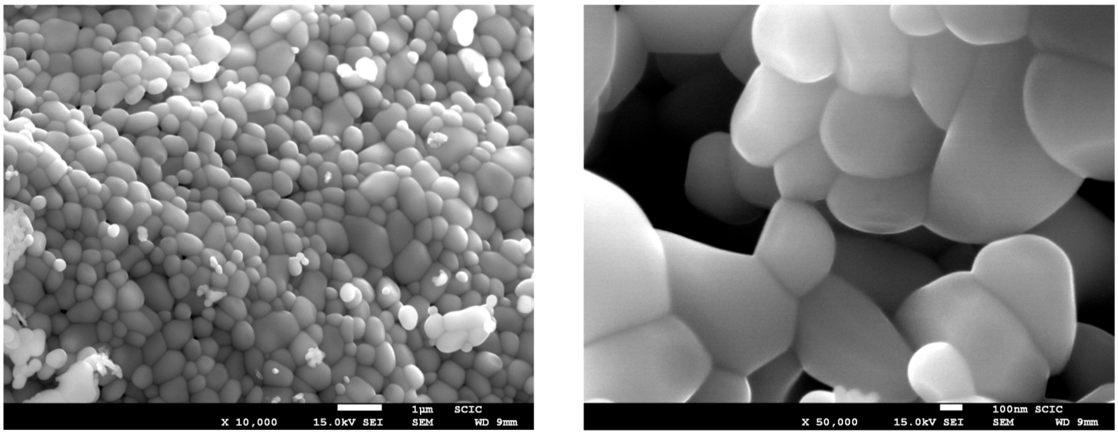

(**a**) (**b**)

Figure S5. FE-SEM images corresponding to non-templated reference $CePO_4$ annealed at 900 °C (under air conditions). (**a**) lower magnification (x 10000); (**b**) higher magnification (x 50000).

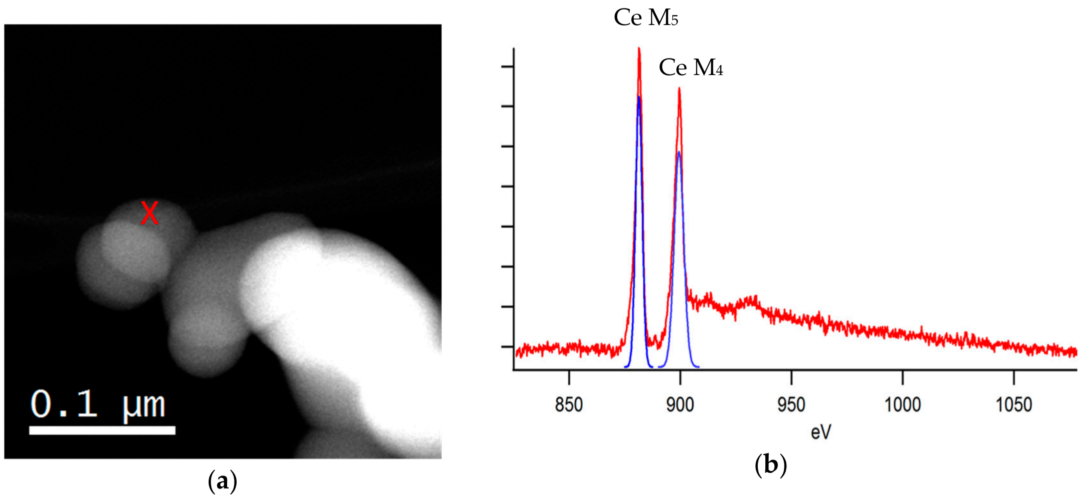

(**a**) (**b**)

Figure S6. STEM-HAADF image (**a**); and corresponding EELS spectrum (**b**) of as-prepared (60 °C-dried) non-templated reference $CePO_4$.
